# Marine Spongin: Naturally Prefabricated 3D Scaffold-Based Biomaterial

**DOI:** 10.3390/md16030088

**Published:** 2018-03-09

**Authors:** Teofil Jesionowski, Małgorzata Norman, Sonia Żółtowska-Aksamitowska, Iaroslav Petrenko, Yvonne Joseph, Hermann Ehrlich

**Affiliations:** 1 Institute of Chemical Technology and Engineering, Faculty of Chemical Technology, Poznan University of Technology, Berdychowo 4, 60965 Poznan, Poland; malgorzata.norman@hotmail.com (M.N.); sonia.zoltowska-aksamitowska@doctorate.put.poznan.pl (S.Ż.-A.); 2 Institute of Experimental Physics, TU Bergakademie Freiberg, Leipziger str. 23, 09559 Freiberg, Germany; iaroslavpetrenko@gmail.com; 3 Institute of Electronics and Sensor Materials, TU Bergakademie Freiberg, Gustav-Zeuner-Str. 3, 09599 Freiberg, Germany; yvonne.joseph@esm.tu-freiberg.de

**Keywords:** marine sponge, spongin, aquaculture, hybrid material, dyes

## Abstract

The biosynthesis, chemistry, structural features and functionality of spongin as a halogenated scleroprotein of keratosan demosponges are still paradigms. This review has the principal goal of providing thorough and comprehensive coverage of spongin as a naturally prefabricated 3D biomaterial with multifaceted applications. The history of spongin’s discovery and use in the form of commercial sponges, including their marine farming strategies, have been analyzed and are discussed here. Physicochemical and material properties of spongin-based scaffolds are also presented. The review also focuses on prospects and trends in applications of spongin for technology, materials science and biomedicine. Special attention is paid to applications in tissue engineering, adsorption of dyes and extreme biomimetics.

## 1. Introduction

**Figure 1:**
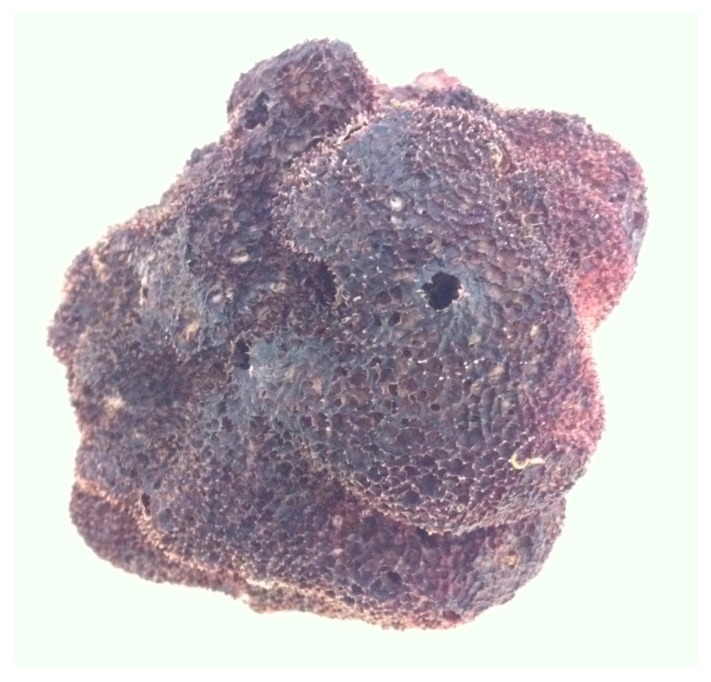
Photo of the typical representative of Dictyoceratida demosponges, known also by the common name “bath sponges”.

**Figure 2:**
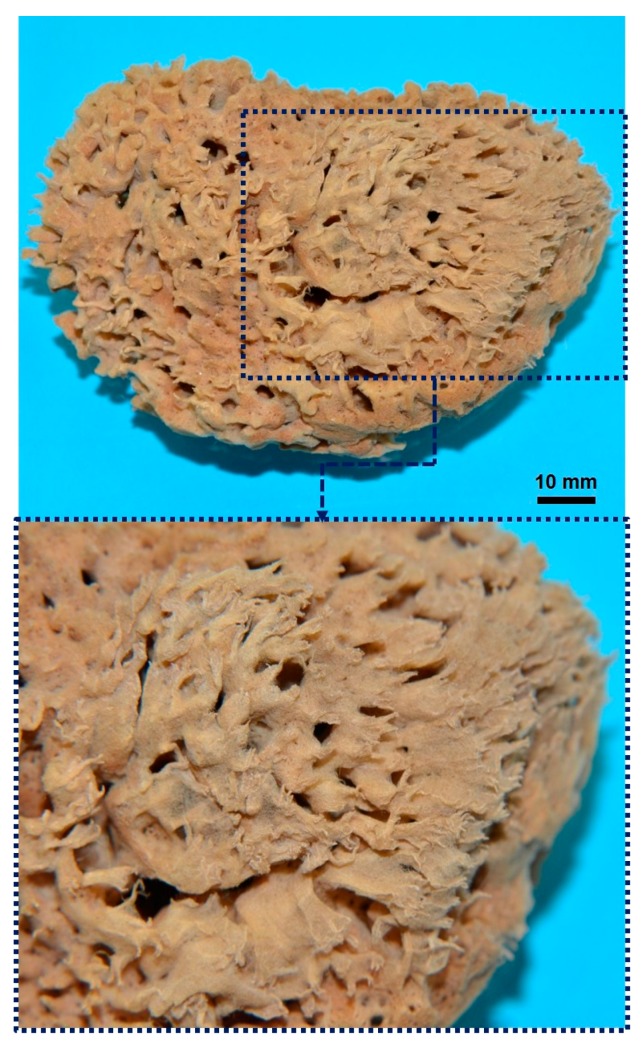
Typical example of a “commercial sponge” after initial bleaching.

Sponges, belonging to the phylum, Porifera, are the phylogenetically oldest multicellular organisms [1], with an evolution dating back to the Precambrian. Currently (2018), there are 8848 valid species described in the World Porifera Database [2], which occur in marine and freshwater habitats. These aquatic animals are currently described in four classes: Demospongiae, Calcarea, Hexactinellida and Homoscleromorpha, based essentially on morphological data and molecular and genetic analyses. Demospongiae is the largest sponge class, including about 80% of all living sponges (nearly 7000 species worldwide), divided into three subclasses: Verongimorpha, Keratosa and Heteroscleromorpha [3]. The focusses of this review are representatives of demosponges which belong exclusively to the subclass, Keratosa, whose mineral-free skeletons consist of “horny” or “keratose” fibers [4,5]. These fibers are known to consist of the halogenated scleroprotein, spongin. Recently the taxonomic name, Keratosa, has been assigned to the group formed by the Dictyoceratida and Dendroceratida orders in the new phylogenetic tree [6,7,8]. Dictyoceratida are defined as sponges with well-developed, anastomosing spongin fiber skeletons, which are hierarchically organized into primary, secondary and sometimes tertiary fibers, and make up a significant proportion of the body volume [9]. Since ancient times, these sponges have been recognized as “bath sponges” [10]. A typical example is the Mediterranean bath sponge *Spongia officinalis*, which is also known as an iconic species with high socioeconomic value [11]. Thus, when we speak about bath sponges, it must be clear that we are referring to the whole organism (body and skeleton) (Figure 1). This is because so-called “marketed sponges” represent cell- and tissue-free, depigmented and demineralized skeletal constructs (Figure 2), which have been defined in the scientific literature as “commercial sponges” [12]. These commercial sponges represent the main source of the “sponge industry” [13].

According to Laubenfels and Storr, “the commercial sponge is the macerated and dried skeleton of one of the sponge animals that has no proper spicules. It must be from a species whose skeleton consists of spongin fibers, and furthermore, these fibers must continue to be elastic or ‘spongy’ even after having been dried” [12]. Traditionally commercial sponges were isolated from diverse representatives of the genera *Spongia* (*S. obliqua, S. officinalis, S. barbara, S. barbara dura, S. anclotea, S. sterea, S. graminea, S. graminea tampa, S. cheiris, S. lamella, S. zimocca*) and *Hippospongia* (*H. gossypina, H. lachne, H. kerion, H. communis*). They differ in fiber morphology, porosity and size. For example, specimens of *S. sterea* of up to 75 centimeters in size have been reported [12].

**Figure 3:**
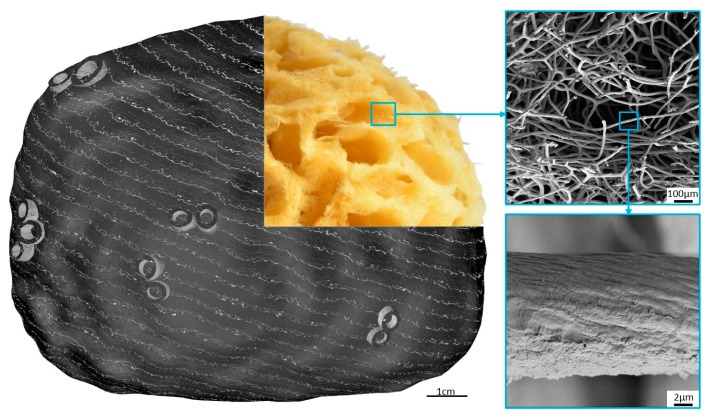
Schematic view of the principal structural motive found in bath sponges. The SEM images to the right represent the morphological features of the spongin-based skeletal construct.

The construct of a commercial sponge consists of a network of spongin fibers which can be from 5 to 100 microns in diameter depending on the sponge species (Figure 3). The meshes are almost rectangular in outline, with dimensions varying from 100 microns to a millimeter.

Although the use of commercial sponges began several thousand years ago, studies on their chemistry with reference to spongin as a biological material date back only to the 18th century. Unfortunately, the pathways of spongin’s biosynthesis in bath sponges as well as the genomics, proteomics and protein sequences of this unique biopolymer, are still unknown. To date, spongin (named also fibrous skeleton, pseudokeratin, neurokeratin, horny protein, collagen-like protein and scleroprotein) has no clear chemical definition [14]. In this review, we focus on the history of commercial sponges, including a brief analysis of the economic aspects of the sponge industry and marine farming of bath sponges as a source for the renewable biomaterial spongin. Next, we examine the structural, physicochemical and mechanical properties of spongin. Special attention is paid to spongin in the form of 3D scaffolds and their applications in tissue engineering, the immobilization of dyes, and the development of novel composite materials using the extreme biomimetics route. We are optimistic that our attempts to establish implications for spongin and the numerous open questions raised in this review will inspire the research community to carry out further, detailed investigations into the chemistry, biochemistry and materials science properties of this evolutionarily ancient structural protein.

## 2. From Commercial Sponges to Modern 3D Spongin Scaffolds: Brief History

**Figure 4:**
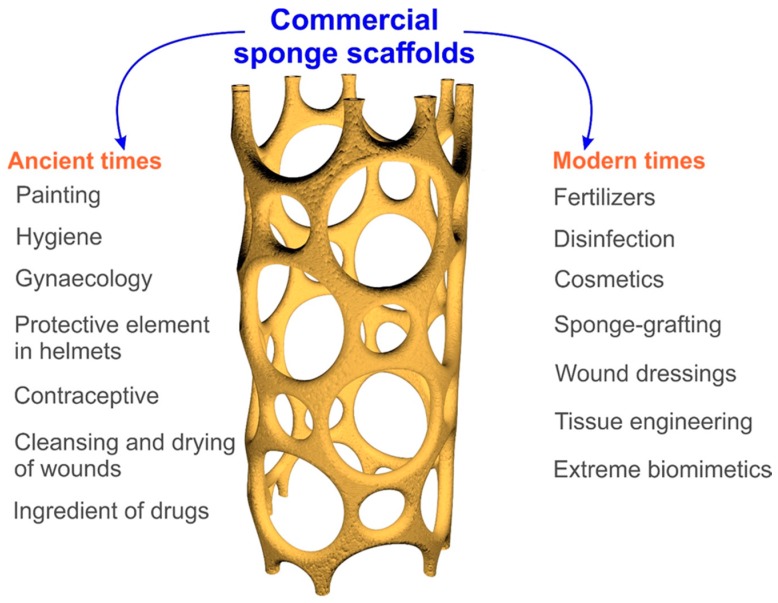
Overview of the diversity of ancient and modern fields of application of commercial sponges.

Commercial sponges were commonly used in ancient times (Figure 4). In the literature, one may find details about the use of sponge skeletons by Egyptian, Phoenician and Minoan civilizations as artistic tools for painting and decoration [15]. Sponges also played a very important role in ancient Greece. Homer’s *Iliad* and *Odyssey*, written around the 8th century BC, contain descriptions of the use of sponges as a hygienic tool [15]. The most accurate data describing applications of commercial sponge skeletons in ancient Greek medicine were collected by Voultsiadou [16]. Sponges squeezed in hot water were used to stop pain in the neck, ears and eyes. Likewise, sponges squeezed in cold water were used to resuscitate people who had fainted, by placing a cold, wet sponge onto the heart—this is the first use of sponges in medicine described by Aristophanes. The wet sponges were also used to counteract heat-stroke by placing them on the head. The ancient Greeks applied honey-filled sponges to treat otorrhea and hemorrhoids or to suckle babies [17]. An indispensable element in ancient surgery, sponge scaffolds soaked in oil were used to fill wounds and for evacuation of nose polypus. They were also used during enemas to block and retain fluids in the anus for a certain period of time. According to Lloyd (1928) [18], the ancient Greeks also used sponge skeletons in gynecology for infections or pains of the uterus, and burned out the skeletons and mixed them with wine to produce a drink used to treat long and intense menstrual periods. They also used burned sponge skeletons as an ingredient of drugs for the treatment of inflammation. Additionally, dry sponge skeletons were used to clean and dry sores, as well as having military uses—for example, to prevent fire in wooden war machines. The ancient Syrians used burned sponges as drug additives to treat laryngeal irritations and coughs [18]. The literature of the Classical Roman age also describes the use of commercial sponge skeletons for washing and personal hygiene [15]. For example, the xylospongium, also known as a sponge on a stick, is the ancient precursor of the modern toilet brush [19]. They were also used by legionnaires of the Roman Empire as a protective element for helmets and as a vessel for drinking water [20]. Sponges are even mentioned in the Bible, in the accounts of the Crucifixion in Matt. 27:48; Mark 15:36; John 19:29 [21].

Intriguingly, the ancient Jews used sponges attached to a string and wrapped in a silk as the most effective contraceptive [15]. For many centuries, sponges soaked in vinegar and lemon juice and plugged inside the vagina before intercourse were employed as barrier contraception. This method was popular until the first half of the 20th century.

In medieval Arabic surgery, sponges soaked with a mixture of hashish, papaver and hyoscyamine juice, dried under the sun and humidified again when required, were placed under the patient’s nose. In the Middle Ages in Europe, sponges were boiled in a brass vessel with a mixture containing specific proportions of opium, hemlock, and the juices of mandragora, ivy and unripe mulberries, until all of the liquid had been soaked up by the sponges. The sponge was then applied to the nostrils of the patient. A sponge full of vinegar was usually applied to the nose to wake the patient after surgery [15].

In the following centuries, the use of sponges did not change significantly. It was not until the turn of the 18th and 19th centuries that a significant breakthrough in the use of commercial sponge skeletons in medicine occurred. Due to the structural properties of commercial sponge scaffolds, they were used as tourniquets to stop bleeding after surgery or tooth extraction, in the treatment of tissue structures and in gynecology for extending the cervix [22,23,24,25,26,27,28]. Burned commercial sponge scaffolds have been reported as drug ingredients for the treatment of sore throats [29]. In traditional European medicine skeletons of the sponges, *S. jodoformiata* and *S. salicylate* were used for disinfection [30]. Moreover, the literature indicates that small fragments of sponge skeletons were applied as prostheses [31]. In the 19th century, due to the good biocompatibility of sponge skeletons, which can act as a support for young vessels, scientists attempted to use commercial sponge scaffolds as a tissue replacement material in the treatment of extensive wounds and swelling [22,26,32]. This process, called sponge-grafting, involved replacement of the wound with a skeleton fragment of an appropriate shape, as a porous substrate for granulating the tissue, until it was completely covered by the epidermis (see for example Hamilton, 1881; Ferguson 1882; Sanctuary 1882; Case 1883; Kendal 1883; Burnett 1885; Acland 1888) [33,34,35,36,37,38,39]. However, problems with the sterilization of sponge skeletons and the very slow or unsuccessful rehabilitation of destroyed tissues led to the abandonment of studies on the use of commercial sponge scaffolds as a filling in tissue defects [40]. Consequently, in the 20th century, they were used for hygienic and cosmetic purposes and to obtain iodine-rich tinctures, which were used in the treatment of various inflammations [41]. Also, at the beginning of the century, air-dried commercial sponges were used as fertilizer by fruit farmers in Key West, Florida [42]. This was a result of the easy availability of commercial sponges at that time, and their chemical composition, which is rich in nitrogen, phosphorus and potassium oxides. In this review we shall discuss some novel directions for the practical application of 3D spongin scaffolds—obtained by further purification of the traditionally used commercial sponges—in tissue engineering, adsorption of dyes and biomimetics.

## 3. The Economics of the Commercial Sponge Industry

Bath sponges were harvested and prepared in the “commercial” form for utilitarian purposes in ancient times by Egyptians, Phoenicians, Greeks and Romans, beginning as early as five thousand years ago [43]. Early Greek literature mentions a well-organized and profitable sponge market [44]. Until the second half of the 19th century, the world’s sponge supply came principally from the Mediterranean Sea [45]. However, beginning in 1841, sponge fisheries were established in the Bahamas (with exports valued at $10,000), in Key West, Florida, and in Cuba. These locations were the leaders in commercial sponge production [45,46]. In the late 19th century, the significant discovery of commercial sponges in the Gulf of Mexico led to growth of a sponge industry in Tarpon Springs, Florida [45]. In the early 20th century (1900–1940) the sponge industry was the most economically important sector in Florida, with a total production of 610,000 pounds. In the same years, Cuba and the Bahamas produced 440,000 and 670,000 pounds respectively [44]. The Florida sponge market was worth more than $1.2 million dollars at that time.

At the beginning of the 20th century the Mediterranean region, especially Greece and Tunisia, produced half of the world sponge supply, valued at $2,390,000. Average annual sponge production in these areas in 1927–1932 was 350 tons per year [47]. The outbreak of World War II, a mysterious sponge disease and overfishing of commercial sponges led to a dramatic fall in production [45,46,48]. Subsequently, all over the world, sponge production declined rapidly, and the value of the US sponge market fell to just $80,000. In the late 20th century, the world sponge supply was dominated by Tunisia (48%), Greece (17%), Cuba (16%) and Florida (19%), with average production amounting to 27–32 metric tons in Florida, 42.5 tons in Cuba and 120 tons for the Mediterranean region [47]. Libya has begun harvesting sponges, with annual production ranging between 4 and 20 tons, but averaging only 5 tons since 2000 [49]. In the last decade, the Mediterranean sponge industry has produced 50 tons of sponges annually [15], with Greece, Tunisia and Libya being the most important producers. Commercial sponge fishing is still an important industry in Egypt, Turkey and the Philippines, although specific data are lacking. In summary, over a long period of time a downward trend in sponge production has been observed; nevertheless, it should be noted that despite the drop in the quantity of sponges harvested, the market is still profitable because of the high price of bath sponges: for example, €10–15 for 1 kg of specimens from the Western Central Atlantic Ocean or €80–100 for 1 kg of sponges from North Africa [49].

## 4. Marine Farming of Bath Sponges and Spongin as Renewable Biomaterial

**Figure 5:**
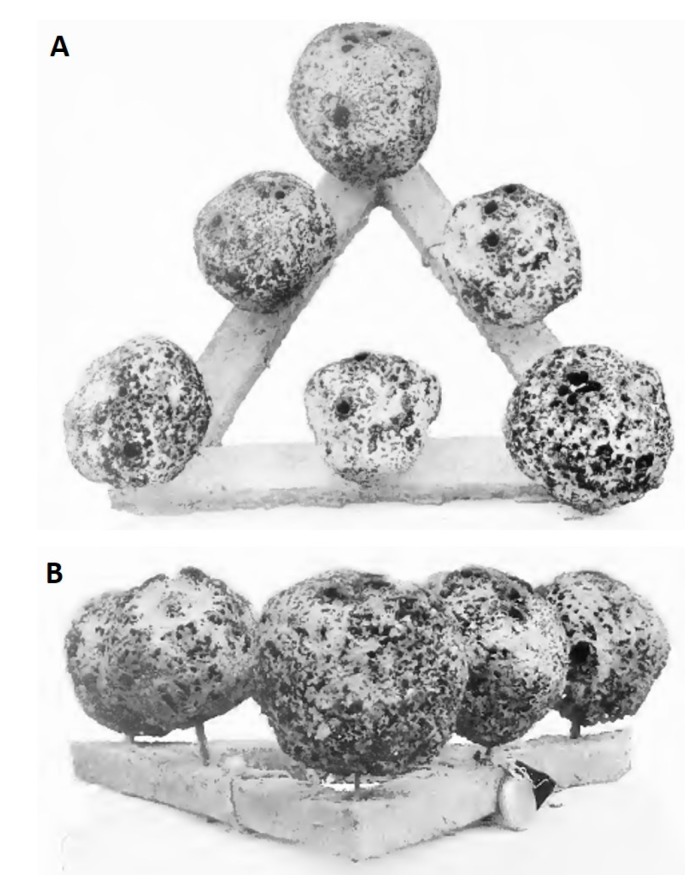
Sponge farming at the beginning of the 20th century: commercial sponges growing on ceramic triangles (**A**, **B**) (adapted from Moore, 1908 [51]).

The renewability of spongin as a biomaterial suggests the need for further development of bath sponge-related marine farming technologies worldwide. The first mention of the possibility of reconstructive growth of commercial sponges is found in a translation of Aristotle’s *Historia* [50]. However, basic information about the cultivation of sponges by cutting was set forth by F. Cavolini as early as 1785 [50,51]. Then, in 1862, Oscar Schmidt made a first attempt to cultivate commercial sponges by the planting of cuttings [50] and obtained several important suggestions for future improvements. Several years later, in 1864, Gregor Buccich carried out research into the farming of commercial sponges at a station on the island of Lesina (Italy) for four years [50,51]. Although his research was not successful, he established several important facts about the growing of bath sponges from cuttings. Full details of this study were described and published by Marenzeller in 1878 [50]. The failure of Buccich’s tests ended the aquaculture of commercial sponges in Europe [51]. At the same time, the idea of cultivating sponges was taken up in Florida. The first efforts were made by Frogaty in 1879, with cuttings attached to wires and stakes [51]. However, these experiments never led to any conclusions, and the results of the study are unknown. At the beginning of the 20th century, Dr. H. F. Moore began experiments at Biscayne Bay to find a way to improve the cultivation of commercial sponges in Florida [50]. He evaluated different methods of sponge farming, including grafting, growing from eggs and planting of cuttings and showed that the use of cuttings was a promising method for cultivating commercial sponges. During his research, he also attempted to identify an optimal method of farming the cuttings, including growing on copper or lead wires and planting on different types of support. After four years of experiments, he concluded that the use of ceramic disks or triangles gave the best results (Figure 5) [51].

In the following decades, the methods described by Moore were modified and improved in different regions of the world [52]. New sponge farms were established in Japan [53,54], Southern Italy [55], France [56], Kalymnos (Greece) [57], the Philippines and Micronesia [58], Australia [59], New Zealand [60] and East Africa (on the Indian Ocean coast) [61]. Some advanced methods of cultivation of bath sponges were described in patents [62,63,64,65]. In summary, sponges have been farmed for more than 200 years using simple methods and cheap equipment, which clearly shows that the cultivation of commercial sponge scaffolds is a cost-effective commercial industry. There are several papers and books providing more detailed information about the aquaculture of commercial sponges [50,51,66].

## 5. Structure, Chemistry, and Properties of Spongin

**Figure 6:**
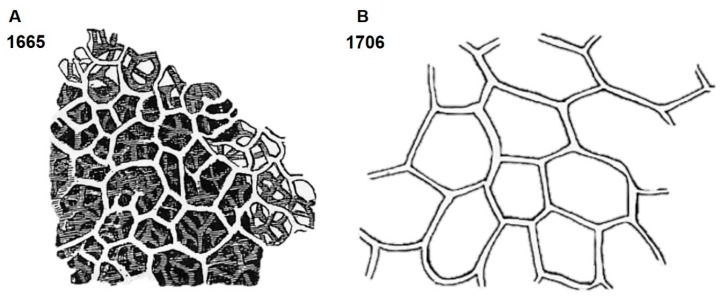
A bath sponge skeleton produced by Hooke in 1665 (**A**) and a simple drawing of the spongin network done by van Leeuwenhoek in 1706 (**B**) (adapted from Arndt, 1931 [68]).

Most authors [67] recognize that the cells which form spongin, the spongioblasts, are derived from the epithelium of sponges. Minchin (1900) [5] claims that the skeletal spongin fibers are secreted to form the large fiber extracellularly. Spongin fibers are large microstructures which have been described for many decades, including with the use of light microscopy. The first drawings of spongin fibers, shown in Figure 6, were made by Robert Hooke in 1665 and by Antonie van Leeuwenhoek in 1706 (see for details Arndt 1931) [68].

**Figure 7:**
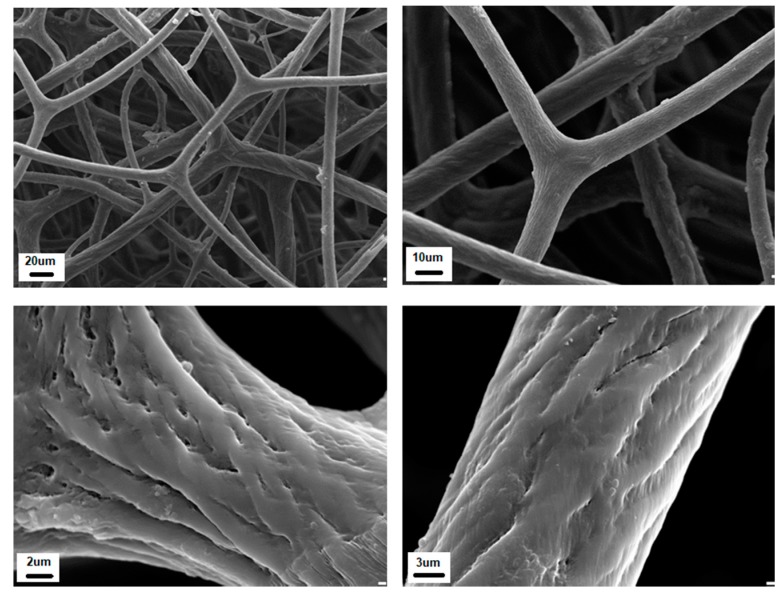
SEM images of *H. communis* spongin skeleton at different magnifications.

The structural features of spongin fibers of different origins observed using electron microscopy have been reported in numerous papers [69,70,71]. According to Garrone (1978) [70], there are two types of fiber structures in the spongin of keratosan sponges: heterogeneous fibers, with a denser surrounding cortical layer and a more granular-appearing medulla; and homogeneous fibers, in which there is no medullary portion. For example, homogeneous fibers containing microfibrils of less than 10 nm in diameter with 65 nm periodicity occur in *S. graminea* [71]. Similar microfibrils within homogeneous spongin fibers can also be observed in the sponge *H. communis* (Figure 7).

In 1841, bath sponges were described as “keratose sponges,” in which the essential base of the skeleton consists of keratose fibrous matter. At that time, the first experiments on the chemistry of spongin were initiated by Croockewit, who suggested a chemical similarity between spongin and silk, although the latter contains no halogen moieties. Croockewit proposed the chemical formula of “horny matter” as 20(C_39_H_62_N_12_O_17_) + I_2_S_3_P_10_ [72]. Due to the identification of iodine within spongin from bath sponges, the term *Jodospongin* was proposed by Hundeshagen in 1895 [73]. Harnack, in 1898, examined ordinary bath sponges which contained 1.1–1.2% iodine, and demonstrated that the halogen must exist in combination with organic components of spongin [74]. During acid hydrolysis it has been found that spongin yields, besides other amino acids, iodogorgonic acid. This is converted into tyrosine and silver iodide by treatment with acid silver nitrate. The yield of iodine is equivalent to 1–2% tyrosine, although 2–8% has been found. This yield is equivalent to 4–7% iodogorgonic acid in the protein [75]. In 1926, Clancey found 14% glycine, 5.7% proline, a trace of cystine, 2.8% tyrosine, 11% tryptophan or histidine, and a remarkably high quantity, 18.4%, of glutamic acid [75]. In 1939, Block and Bolling presented results on the chemistry of spongin and reported the content of glycine as about 14% [76]. This is much lower than the concentration of glycine in collagen, which is between 25 and 33% [77]. Consequently, spongin from bath sponges was recognized, for the most part, as a halogenated scleroprotein or neurokeratin-like protein [76] due to the presence of cystine.

Spongin chemistry is thus found to be extremely complex, due to the presence of diverse halogens (I, Br) which have never been reported in natural collagens or keratins. This may explain the very high resistance of this structural protein to enzymatic treatment. Spongins have been characterized by numerous researchers as being especially resistant to various enzymes, including proteases, trypsin, pronase, collagenases, amylases and lysozymes [69,78]. On the other hand, in a natural environment, diverse microorganisms are able to destroy spongin enzymatically and cause very high levels of damage to the structure of the spongin-based skeletal fibers (for details see Gaino et al. [79]). The isolation and purification of such special “sponginases” remain a challenge for future research and will be important methods for obtaining peptides, which will be useful for detailed proteomic analysis and the sequencing of spongin.

**Figure 8:**
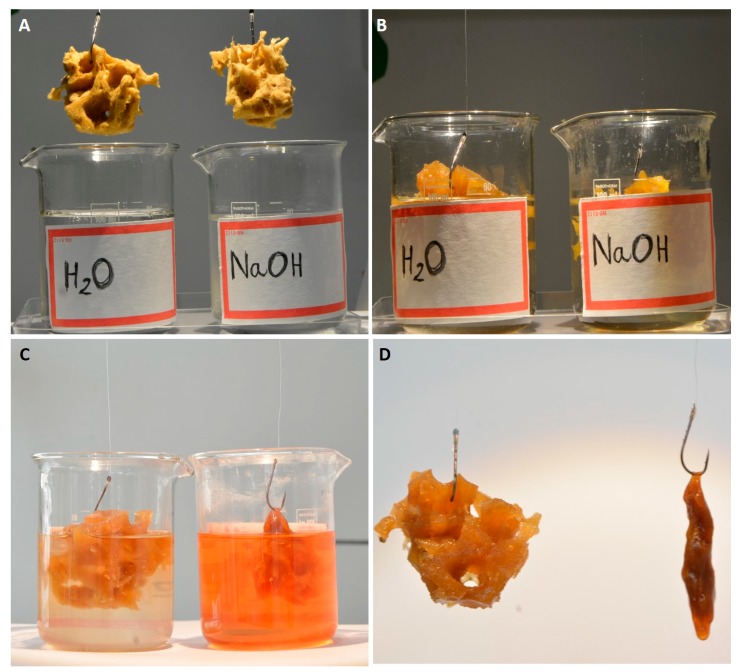
Selected fragments of spongin scaffolds isolated from *H. communis* demosponge (**A**) were placed in vessels with deionized water and 5% NaOH solution (**B**) at room temperature. Structural changes became visible after 48 h of treatment with an alkaline solution (**C**). Finally, the spongin scaffold lost its 3D architecture and structural integrity (**D**). See also Figure 9.

**Figure 9:**
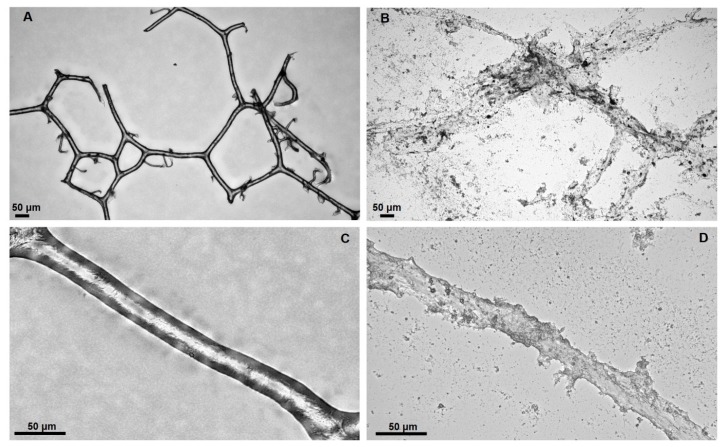
Disintegration of spongin from *H. communis* (**A**,**C**) using 5% NaOH (**B**,**D**) at room temperature. Images **B** and **D** correspond to the state of the spongin scaffold shown in Figure 8D.

The physicochemical properties of bath sponge spongin are important for its practical applications. Spongin is insoluble in water and acids. The fibers withstand treatment with 3M HCl and 5% trichloroacetic acid at 90 °C [80]. Detailed information about the influence of diverse chemical reagents on spongin can be found in patented literature related to the bleaching or dyeing of commercial sponges [81,82,83]. For example, it was reported [84] that commercial sponge scaffold cannot be dissolved in sulfuric acid/hydrogen peroxide/sodium bisulfite solutions. Also, the addition of ammonia does not dissolve this material. On bleaching of sponge using sodium permanganate, the oxide, MnO_2_, forms on the surface of the sponge—it may be removed by treatment with sodium bisulfite [84]. On the other hand, alkalis can dissolve the spongin material into hydrolysates of amino acids. The procedure of alkali treatment on spongin-based scaffolds is easy to observe (see Figure 8). The dissolution of spongin fibers is also easily visible under white light microscopy (Figure 9).

## 6. Material Properties of 3D Spongin Scaffolds

It has long been recognized that living bath sponges are commonly elastic, owing to the spongin skeleton [85]. Of commercial sponges, those which have soft, durable, absorbent, and elastic fibers are the most expensive [66,86]. Consequently, the basic desirable qualities of a sponge—ability to hold water, compressibility, resiliency, and toughness—are all dependent upon its spongin fiber pattern and structure [13]. The structure-function relationship in spongin-based scaffolds is based on both the pattern and size of the fibers. We recall here some postulates on the structural properties of skeletal fibers of commercial sponges proposed by von Lendenfeld in 1889:

“1. Compressibility of the sponges is partly dependent upon the shape of the meshwork. The more regularly polygonal the meshes, the harder the sponge. The greater number of simple branching of the fibers, the softer and more absorbent the sponge.

2. The sponges with connecting fibers of about 0.02 mm thickness appear to be the most elastic. The thicker the fiber, the more rigid the sponge. Finer the fibers, softer the sponge.

3. If any of the fibers of the sponge contain foreign bodies, these fibers are readily crushed when the sponge is compressed, and the sponge loses its elasticity.

4. The more numerous the fibers per unit of volume, the greater the capillary action and the more water the sponge can hold” [87].

From the standpoint of materials science, commercial sponge can be characterized by its porosity, flexibility, and compressibility. There is no doubt that such material parameters as softness, fineness, absorptiveness, toughness, elasticity, and durability of spongin-based 3D constructs are species-dependent [13]. For example, the average tensile strength of the Mediterranean Elephant Ear sponge (*Agelas clathrodes*) was found to be 2.88 ± 0.19 kg·cm^−2^, and that of the Philippine Elephant Ear sponge, 6.88 ± 0.77 kg·cm^−2^; the corresponding values for elasticity were 26.1 ± 0.79% and 7.8 ± 0.6% [88]. It was suggested that the differences are related to the structure and arrangement of the spongin fibers. The orientation of the secondary fibers with a very compact and regular structure, as well as the abundance and the arrangement of the primary fibers in the form of bars perpendicularly oriented to the external surface, give the Philippine Elephant Ear sponge a higher tensile strength, a roughness to the touch, and consequently, inelasticity and stiffness [88].

**Figure 10:**
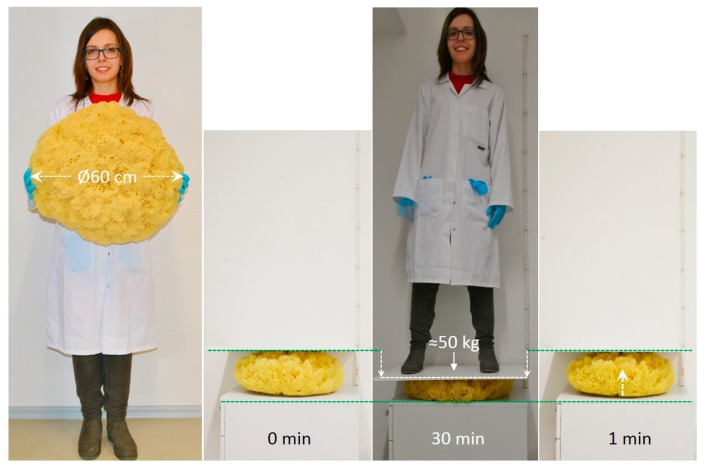
Demonstration of the resistance of commercial sponge to mechanical pressure over 30 min and the ability to recover its initial form and volume in 1 min.

The network structure of spongin fibers determines one of the most attractive features of commercial sponges—their compressibility [89]. A simple test demonstrating the resistance to strain under pressure and the flexibility of a commercial bath sponge of the species *H. communis* is presented in Figure 10.

This experiment clearly indicates that a large spongin-based scaffold of up to 60 cm in diameter is elastic and flexible when considering its resistance to compression. After being compressed under a weight of 50 kg, the scaffold returns to its original shape and volume in 1 min (Figure 10).

In modern times, the mechanical properties of commercial sponges are important in the design of further applications. For this purpose, a number of mechanical parameters, such as firmness, compression modulus, compressive strength, tensile strength, elastic limit, elastic strain, modulus of elasticity, and modulus of resilience must be measured. An attempt to develop a comparative test was made by Louden et al. [20]. They analyzed several species of marine demosponges, including three commercial species—*H. lachne*, *S. graminea*, and *S. zimocca*—and showed that selected commercial sponges differ in mechanical properties, such as strength, softness and elasticity, which creates a unique profile for each species. It was shown that measurement of the elastic modulus enables the selection of suitable sponges for applications in cosmetics and medicine. Additionally, the correlation between the density and mechanical parameters makes it possible to refine the tests and more accurately determine the usefulness of commercial sponge scaffolds.

## 7. 3D Spongin Scaffolds and Tissue Engineering

Over the millions of years of their evolution, keratosan demosponges have developed a unique strategy to support their life as sessile organisms which need to pump huge amounts of seawater, filtering it to capture food in the form of diverse organic microparticles. Both the demosponge skeleton and body are designed for efficient filtration of the surrounding seawater as a food source. The porous 3D architecture of mechanically rigid, spongin-based sponge skeleton is efficiently constructed to favor the survival of the organism. There is no doubt that the fibrous architecture and porosity of spongin-based skeletons in keratosan demosponges produce a naturally occurring superior scaffolding for diverse cells which are crucial for the sponge as an organism. On the other hand, the present generation of tissue engineering research is based on the seeding of appropriate cells onto porous biodegradable polymer matrices [90]. At this point, the paths of sponges and tissue engineers cross, especially with regard to similarities in design concepts. Tissue engineering involves a search for biomaterials which can serve as temporary matrices, while sponges provide examples of naturally “prefabricated”, practically ready-to-use 3D scaffolds (see Figure 2 and Figure 3). As the processing of artificial scaffolds (even using biopolymers, such as chitosan, collagen and gelatin) is technologically difficult and expensive, such spongin-based scaffolds may be of interest due to the possibility of generating the required amounts of these materials from natural sources, for example marine ranching or cultivation of spongin-based keratosan sponges (see Section 4 for details).

Interestingly, successful attempts to use spongin in the form of commercial sponges as biomedical implants have been reported since the 18th century. Fragments of the bath sponge skeleton were used as small prostheses in early “plastic surgery” [31]. Revolutionary results can be found in the paper published by Hamilton in 1881 under the title “*On sponge-grafting*” [33]. In the reported case, a woman underwent surgery for the removal of a mammary tumor, during which a large area of skin was removed. This skin was replaced with a thin slice of aseptic sponge skeleton, which, ten days after the surgery, was observed to be vascular, and three months later, was covered with epithelial tissue [33].

Today, the biocomposite structuring, mechanical design and design of porous interfaces on nano- and micro-levels of both chitin-based [91,92] and spongin-based [93,94,95] demosponge scaffolds are recognized as applicable for the requirements of modern tissue engineering. Optimistic results have been reported with human osteoprogenitor cells on the skeleton of *S. officinalis* [93], with osteoblast-like MG-63 cells growing on spongin from *Hymeniacidon sinapium* [14], and with mouse primary osteoblasts on spongin from *Callyspongiidae* marine demosponges [96]. Spongin from the marine sponge, *Biemna* sp., alone and in combination with growth factors, has been recently shown to be a promising biomaterial for bone augmentation and bone repair [97].

## 8. 3D Spongin Scaffolds as a Support for Dye Immobilization

**Figure 11:**
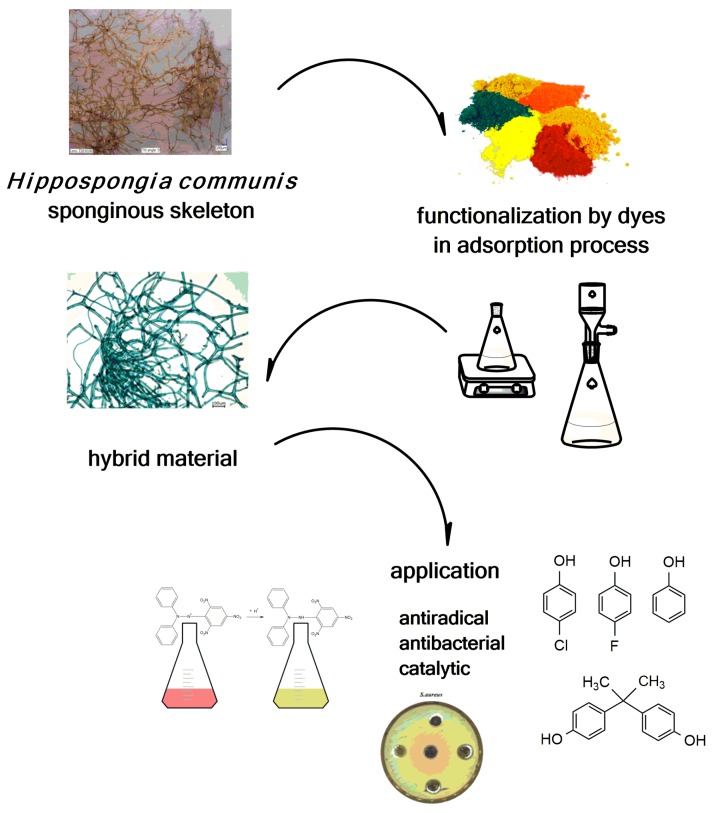
Scheme of *H. communis* spongin scaffold functionalization using dyes, and further applications of the produced hybrid material.

Literature reports emphasize the scientific and applicative potential of marine keratosan sponges. Moreover, the sponges’ wide range of properties enables further functionalization of selected marine demosponge skeletons as special scaffolds to improve their surface properties and enable their use in various new areas. The functionalization of *H. communis* spongin skeletons has been carried out using dyes. Novel dye/biopolymer hybrid materials with designed properties combine the beneficial features of both constituents (Figure 11).

Dyes occurring in nature were the only dyes available to mankind for use in coloring until the invention of the first synthetic dye in the 19th century. Natural dyes are derived from plants, animals, microbes or minerals. Natural dyes from plant and animal sources have been used by people for centuries. In modern times many such compounds have been scientifically studied, and new information about their anti-cancer, anti-inflammatory and antioxidant properties has been published [98,99,100,101]. Natural colors have some disadvantages compared with synthetic dyes, as they are more expensive and less stable, being susceptible to degradation by light, pH, temperature, sulfites, ascorbic acid and enzymes [102]. Natural dyes are renewable and sustainable bioresource products with minimum environmental impact; they are biodegradable and non-toxic [103]. They have applications in food and textile coloration, as pH indicators, in cosmetics, in pharmaceuticals, and in dye-sensitized solar cell production [104]. However, the most important limitations reported are their low bio-distribution and bioavailability, as well as instability. Therefore, adsorption of these natural compounds onto biopolymer supports can be expected to improve their stability and enable exploitation of their antibacterial and antiradical properties in new applications.

In one study [105] concerning dye adsorption onto sponginous scaffolds, carmine—an anthraquinone dye—was chosen as an adsorbate. The results of physicochemical tests confirmed the sorption properties of spongin and its affinity to dyes. The adsorption process was fast and facile, and the key parameter affecting its efficiency was pH: an acidic solution promotes adsorption. Analysis of FTIR and Raman spectra suggests that the spongin scaffold interacts with carmine by hydrogen bond formation between the -OH and -COOH groups of the dye and the marine sponge skeleton. The results of this study open up new possibilities for the synthesis of dye/spongin hybrid materials, which are of particular interest in the development of cosmetics, wound dressing and drug delivery systems.

A considerable part of the research consisted of evaluation of the practical properties of the obtained materials and their further application. One of the objectives of the study was the combination and utilization of the beneficial properties of sponginous scaffolds isolated from skeletons of *H. communis* and selected dyes. Because of its nontoxicity and antiradical properties, naturally occurring anthocyanin has potential for utilization as a harmless coloring material. Its color instability, for example, under light irradiation, limits its practical use. Adsorbed anthocyanin exhibits antioxidant properties comparable to those of a dye solution. This may provide a better chemical system in terms of stability, without loss of bioavailability. In another study [106], a system was obtained with natural-anthocyanin dyes adsorbed on spongin scaffold. It was found to have antioxidant properties, using a modified version of the Brand-Williams method (reduction of the DPPH 1,1-diphenyl-2-picrylhydrazyl radical to DPPH-H: 1,1-diphenyl-2-picrylhydrazine). A radical scavenging yield of more than 95% was obtained after 30 min of the process. The Trolox equivalent was calculated to compare the results with others previously reported. The developed spongin hybrid materials were comprehensively analyzed using Fourier transform infrared spectroscopy (FTIR), cross polarization magic angle spinning nuclear magnetic resonance (^13^C CP MAS NMR), thermogravimetry (TG), elemental analysis (EA), and optical and scanning electron microscopy (SEM). The analyses indirectly confirmed the effective adsorption of anthocyanin onto the sponginous scaffold.

A number of forms of bioactivity are ascribed to chlorophyllin, the dye used as an adsorbate in a further study [107]. Chlorophyllin is known to suppress the mutagenic and carcinogenic effects of compounds having polycyclic structures, such as heterocyclic amines and aflatoxin B. In a report by Kobayashi et al. [108] a chlorophyllin/chitosan system was produced as a trap for polycyclic mutagenic compounds. Similar results were described in a publication by Hayatsu et al. [109]. Chlorophyllin adsorbed on a copper-containing hydrotalcite was found to be most effective in the bactericidal treatment of industrial wastewater against *Escherichia coli*, *Enterobacter aerogenes*, *Salmonella enterica*, and *Staphylococcus aureus*, as reported by Oliveira et al. [110]. To enhance the antimicrobial effect of graphene oxide against *E. coli*, water-soluble chlorophyllin was used to prepare functionalized graphene oxide nanomaterials [111].

Knowledge of the antibacterial properties of chlorophyllin led to a decision to test it in combination with spongin scaffolds. The antibacterial activity was evaluated based on the diameter of the zone in which growth of the selected bacteria strain was inhibited. The results demonstrate that the marine sponge skeleton/chlorophyllin hybrid material reduced the growth of the Gram-positive microorganism, *S. aureus*, and the effect increased with an increased concentration of SCC in the hybrid material, as expected. The basic advantage of the obtained system in comparison with the pure dye is its insolubility, which provides the possibility of reuse. This is important in terms of its potential applications, which include the preparation of wound dressing. There are reports concerning the use of sponges, both synthetic—polyurethane, poly(vinyl alcohol), poly(e-caprolactone)—and natural (*Luffa cylindrica*), in wound management to promote healing of acute and chronic wounds [112,113,114]. For example, negative pressure wound therapy is a dynamic wound closure system that uses topical, controlled negative pressure continuously or intermittently in wound management. A vacuum is used to reduce pressure around the wound, to draw out excess fluids and cellular wastes, and to promote the formation of granulation tissue [115]. To maintain the vacuum and the moist wound setting, the defect is sealed with a sponge. The same study pointed out the risk of *S. aureus* infection [116]. It is significant that the *S. aureus* strain is a bacterium responsible for hospital-acquired infection.

The favorable results obtained for chlorophyllin adsorption provided motivation for further studies using other dyes with a porphyrin-like structure. One group of such dyes is the phthalocyanines. Because of their structure, they exhibit unique properties: redox activity, high molar absorption coefficient, high thermal stability and reactivity, as well as high resistance to oxidants, acids and alkalis; these enable the use of phthalocyanines in photodynamic therapy [117], electronics [118,119], sensors [120,121,122], dyes and solar cell production [123,124,125]. Metalphthalocyanines have been used as efficient biomimetic catalysts for oxidation, reduction and other reactions of organic compounds. Metalphthalocyanines have demonstrated high catalytic activity under ambient conditions, as well as good resistance to oxidants, acids and alkalis. Immobilization of these compounds on a support is an effective method for heterogeneous catalyst production. Such a system has several advantages over homogeneous types: the possibility of reutilization, higher surface area, and better access to active sites. Adsorbents used to date include zeolites [126], silica [127], TiO_2_ [128], carbon-based materials [129,130], polymers [131] and fabrics [132].

**Figure 12:**
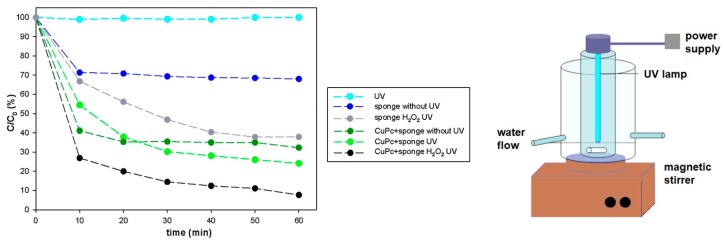
Concentration of Rhodamine B as a function of time under various experimental conditions, and a schematic diagram of the UV reactor.

In a new approach, copper phthalocyanine was adsorbed onto marine sponge skeleton and used in the catalytic degradation of the synthetic dye, Rhodamine B [133]. The results confirmed that the catalytic properties of copper phthalocyanine are retained in combination with spongin, and that this biomaterial is a good support for the catalyst. In this study a combined approach for Rhodamine B degradation was used: photochemical systems based on photosensitizers absorbing UV light (CuPC) which function as catalysts, and an external oxidant H_2_O_2_ which forms strong oxidizing species like OH∙ that react directly with the molecules of Rhodamine B. The synergistic effect of these factors led to rapid and almost complete (up to 95%) decomposition of Rhodamine B (Figure 12).

On the FTIR spectra of the hybrid material obtained from the adsorption process, bands originating both from the spongin matrix and the dyes are observed. The most important changes relate to the stretching asymmetric vibrations of sulfonic groups (−SO^3−^M^+^) occurring in the wavenumber range, 1250–1140 cm^−1^, in the form of a broad band observed in the spectra of spongin and of the product obtained by adsorption of the above-mentioned dyes.

The promising results obtained for copper phthalocyanine provided motivation to test another metalphthalocyanine in a catalytic process. In a further study [134] the catalytic performance of an iron phthalocyanine/spongin scaffold hybrid material was evaluated. The common aromatic water pollutant phenol, and its halogenated derivatives chloro- and fluorophenol as well as bisphenol A (BPA), were taken as substances for degradation.

In the study by Normal et al. [134] the effects of time, presence of hydrogen peroxide, ultraviolet irradiation, adsorption and catalyst addition on degradation efficiency were evaluated. Similar to the previously mentioned publication [133], the best results were obtained when all of these factors worked together. The synergistic effect was calculated, as well as the degradation kinetics according to pseudo-first-order and pseudo-second-order models. A significant part of the work consisted of the identification of degradation intermediates and products. Based on the products identified by high-performance liquid chromatography/mass spectrometry (HPLC-MS) and on the basis of the existing literature, a possible mechanism and pathways of degradation were proposed, featuring a series of steps, including cleavage of C–C bonds and oxidation.

X-ray photoelectron spectroscopy (XPS) was used to determine the exact chemical states of the species on the surface of the spongin. The recorded binding energies for spongin suggest the presence of C–H (~283 eV); C–C, C=C, C–OH (~285 eV); N–C=N, C–O–C (~286 eV) and C=O (N–C=O, O–C=O) (~288 eV) bonds in its structure. After adsorption of iron phthalocyanine, besides the elements carbon, oxygen and nitrogen, iron and sulfur were detected on the surface of the iron phthalocyanine/biopolymer hybrid, and changes in binding energies were observed, occurring especially at the sulfonated group sites.

Dyeing of marine sponges has been previously described in certain patents [82]. However, the dyeing conditions presented there are radical; the process is multi-staged and includes preliminary washing, bleaching, stain removal, color precipitation and development. The above-mentioned publications confirm the promising properties of spongin skeleton as a dye adsorbent, requiring no special preparation of the sponge and enabling the adsorption process to be carried out under mild conditions.

The preparation of sponge skeleton/dye systems makes it possible to combine the functional properties of selected dyes with a thermally and mechanically resistant support of natural origin, and to create products with unique physicochemical properties and the potential for interesting applications. Moreover, the benefits of the 3D spongin scaffold, such as its three-dimensional, anastomosed architecture, affinity to different structures of dyes and high sorption capacity, make this material a promising candidate for use as a catalyst support.

## 9. Spongin and Extreme Biomimetics

Extreme Biomimetics is a novel field in modern bioinspired materials science, established in 2010, in Germany [135]. This research field has developed a new way of thinking about the next generation of biologically inspired composites with properties that will allow their applications in the extremes of modern industry. The approach is based on the functionalization of thermostable biopolymers using inorganic compounds under conditions that imitate natural processes occurring at extremely low or extremely high temperatures, as well as processes taking place at high pressures, salinity and pH levels (for a review see Ehrlich ed. 2017) [136]. For example, Ehrlich et al. used an extreme biomimetic strategy to develop a zirconium oxide phase on the surface of chitin, which is thermostable up to 400 °C [91]. This approach has also been used to create novel multiphase composites, such as chitin/zirconia [90], β-chitin/ZnO [137], chitin/GeO_2_ [138], chitin/hematite [139,140], chitin/(Ti,Zr)O_2_ [141], chitin/silica [142,143] and chitin/POSS [144], for various applications. Recently, an extreme biomimetic route was also used for the first time for the development of pectin/GeO_2_ composites [145].

**Figure 13:**
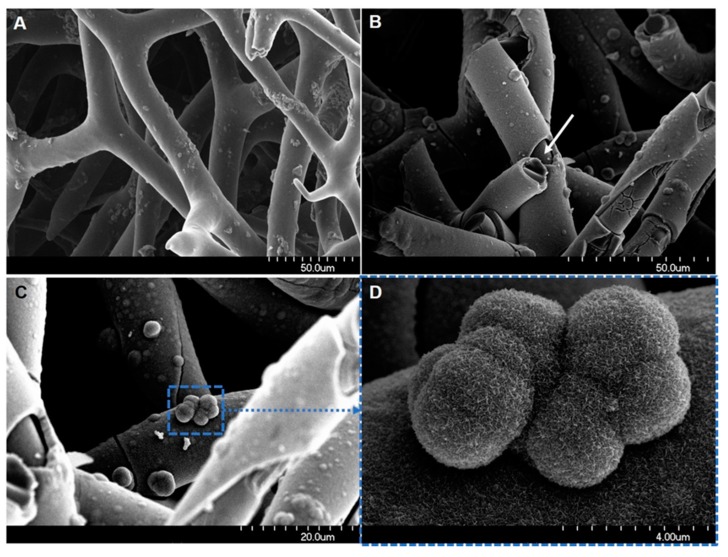
Functionalization of spongin fibers for the development of a 3D structured catalyst. The spongin fibers (**A**) can be effectively carbonized under a nitrogen-rich atmosphere and tightly covered with a manganese oxide layer (**B**). The carbonized spongin became visible only on the mechanically damaged surface, with the surface of the fibers covered with the oxide (arrow). The formation of a secondary Mn oxide nanocrystalline phase can also be observed (**C**, **D**).

As spongin is thermostable up to 260 °C [146,147], several successful attempts to use this structural protein as a scaffold under an extreme biomimetic approach have been recently reported. Szatkowski et al. produced an Fe_2_O_3_/spongin-based composite at 90 °C, obtaining a material that had a positive effect on the capacitance of energy storage devices [146]. In further work, the same team immobilized TiO_2_ on 3D spongin scaffolds at 120 °C and used the resulting composite as a photocatalyst for removal of the dyes, C.I. Basic Blue 9 [147] and Methylene Blue [148], obtaining satisfying results for both sorption and photocatalytic processes. At present, a new trend in the extreme biomimetic field is the design of 3D spongin-based scaffolds formed at temperatures over 500 °C in an oxygen-free atmosphere. An example is a recent study by Professor Jesionowski’s group [149] in which a novel MnO_2_/carbonized spongin composite was synthesized. The 3D structured composite features the unique network of spongin (maintained even after carbonization at 650 °C) and well-defined nanostructured MnO_2_ (Figure 13). This novel composite exhibits excellent electrochemical properties and was stable over more than 3000 charging/discharging cycles in a cyclic voltammetry experiment.

## 10. Conclusions

In contrast to such structural proteins as collagen, fibroin (silk), elastin, resilin and keratin, the chemistry of spongins and their molecular biology, including sequences, so far remain unknown. It would appear that spongin is the last enigmatic proteinaceous biopolymer, even though it is of very ancient origin and has undergone more than 300 years of chemical and structural investigations. Fundamental questions about the pathways of keratosan spongin biosynthesis and its relationship with key biochemical reactions involved in the biosynthesis of both collagen and keratin are still open. There is a lack of knowledge concerning the mechanisms of spongin halogenization under natural conditions. What are the functional roles of iodine, bromine and sulfur within spongin? Also, the challenging task of isolating and identifying specific spongin-degrading proteases still remains to be addressed. The mechanisms of microbial digestion of spongin in living, but damaged, bath sponges ought to be investigated. It would seem that after thousands of years of usage of commercial sponges, mostly as cosmetic tools, it is now time to find new applications for them in novel technologies, including the large-scale desalinization of sea water, adsorption of radionuclides, and remediation of diverse hydrocarbon pollutants.
